# The surgical strategy for multilevel massive ossification of the posterior longitudinal ligaments

**DOI:** 10.3389/fsurg.2022.1066120

**Published:** 2022-12-23

**Authors:** Ying-Ching Li, Zhuo-Hao Liu, Ying-Sheng Li, Mun-Chun Yeap, Yu-Tse Liu, Yin-Cheng Huang, Ching-Chang Chen, Po-Hsun Tu

**Affiliations:** ^1^ Department of Neurosurgery, Chang Gung Memorial Hospital at Linkou, Chang Gung Medical College and University, Taoyuan, Taiwan; ^2^ School of Medicine, National Tsing Hua University, Hsinchu, Taiwan; ^3^ Division of Thoracic and Cardiovascular Surgery, Department of Surgery, Chang Gung Memorial Hospital at Linkou, Chang Gung University, Taoyuan, Taiwan

**Keywords:** cervical myelopathy, laminectomy, outcome, ossification of the posterior longitudinal ligaments, anterior decompression and fixation

## Abstract

**Purpose:**

Creating enough decompression, favorable outcome, less complication, and maintain adequate lordosis and stability in the patients with cervical myelopathy due to multilevel massive ossification of the posterior longitudinal ligament (OPLL) still poses a challenge for surgeons. The aim of our study is to retrospectively evaluate our patients and try to seek a better surgical strategy.

**Methods:**

Between 2015 and 2019, 55 consecutive patients with multilevel massive OPLL underwent surgical treatment. Among these, 40 patients were treated with cervical laminectomy and then anterior decompression, fusion, and fixation (ADF), which was defined as group 1, and 15 patients were treated with cervical laminectomy and fixation simultaneously, which was defined as group 2. The patient's radiographic characteristics and postoperative outcomes were evaluated.

**Results:**

Better postoperative cervical sagittal lordosis and less long-term axial pain was achieved in group 1 (*p* < 0.001), though the functional outcome had no significant difference. In the multivariable analysis, anterior fixation accounts for independent factors for better cervical sagittal alignment (*p* < 0.001). No complications directly associated with cervical laminectomy were observed.

**Conclusion:**

In patients with cervical multilevel massive OPLL, laminectomy at compression level and then ADF depended on the severity and range of compression, but corpectomy of not more than two vertebral bodies is suggested, except K-line (+) and long-segment massive OPLL majorly involving the C2 and posterior laminectomy above and below the OPLL-affected levels with posterior fixation simultaneously.

## Introduction

Ossification of the posterior longitudinal ligament (OPLL) is also an important pathology and is not a rare degenerative spine disease that causes neurologic dysfunction in middle-aged and elderly patients (1). As OPLL develops, patients with progressive degenerative cervical myelopathy (CM) require surgical treatment (2, 3). Surgical alternatives include anterior, posterior, or combined anterior and posterior decompression and/or stabilization. However, the optimal surgical procedure for the treatment of cervical OPLL is a matter of debate, especially for multilevel compression and massive OPLL with a more than 60% canal occupying ratio (COR) (4). Posterior approach includes laminectomy, laminoplasty, or laminectomy with fusion and fixation, is relatively simple, allows easier decompression of multiple levels, and has a low complication rate (5–10). However, the effect of indirect decompression of the spinal cord by posterior decompression is limited for patients with severe kyphotic deformity and/or massive OPLL (2, 3, 11–14). Even more, posterior laminoplasty in patients who have massive OPLL with a ≥50% COR can result in poorer outcomes compared with anterior decompression and fusion with fixation (ADF) (15, 16). ADF, including cervical multilevel corpectomy or discectomy, can provide direct decompression to the spinal cord, can maintain suitable alignment of the spine, and can stabilize the involved segments (2, 3, 17). Several studies have reported that the postoperative neurologic recovery rate is similar in patients who accepted ADF and posterior decompression and fusion with fixation (PDF), but ADF is superior to PDF in patients with kyphotic alignment and higher COR. The advantages of ADF include better radiographic outcomes and faster neurological recovery rates (2, 11, 15). However, the ADF has a high complication rate (18, 19), is technically demanding, and has limitations in cases with multilevel OPLL or OPLL majorly involving the C2. In a recent review, ADF is considered for K-line (−) regardless of canal occupying ratio, and K-line (+) and COR >60% for OPLL patients. Posterior approach is considered for patients with multilevel compressive myelopathy, K-line (+), and COR < 50%–60% for OPLL (20, 21). However, there is higher graft dislodgement to perform multilevel ADF (22), poor decompression effect on OPLL majorly involving the C2, and relative difficulty in correcting the kyphotic alignment for performing PDF in these OPLL patients. Although posterior laminectomy, ADF, and then posterior fixation could provide safe decompression, cervical realignment, and favorable outcomes, and less long-term complication in the patients with cervical multilevel OPLL with kyphotic deformity and higher COR (23, 24), little case numbers and higher short-term morbidity is still worth to be concerned. Whether there is the simpler but still safe and effective surgical strategy for cervical multilevel OPLL with kyphotic deformity and higher COR.

In this study, we analyzed our cases with cervical multilevel OPLL with kyphotic deformity and higher COR, compared the functional outcomes and complication between laminectomy with ADF and PDF, and try to seek a better surgical strategy.

## Material and method

### Patients

**Figure 1:**
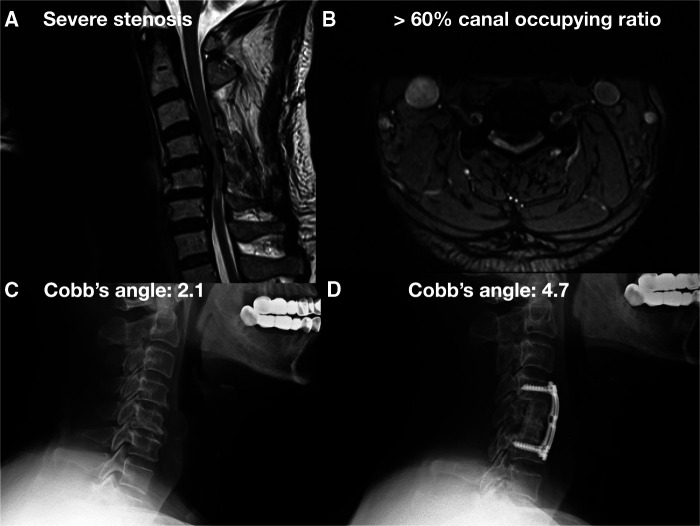
A 63-year-old male with CM due to long-segment massive OPLL over C3-6 underwent C3-6 laminectomy followed by anterior C4-5 and C5-6 microdiscectomy and C5 corpectomy, and C4-6 interbody fusion and internal fixation. (**A**) Preoperative cervical MRI showed severe cervical stenosis with bulging disc. (**B**) Preoperative cervical MRI axial view showed >60% canal occupying ratio. (**C**) Preoperative cervical x-ray showed cervical lordosis about 2.1°. (**D**) Postoperative cervical x-ray showed cervical lordosis about 4.7°.

**Figure 2:**
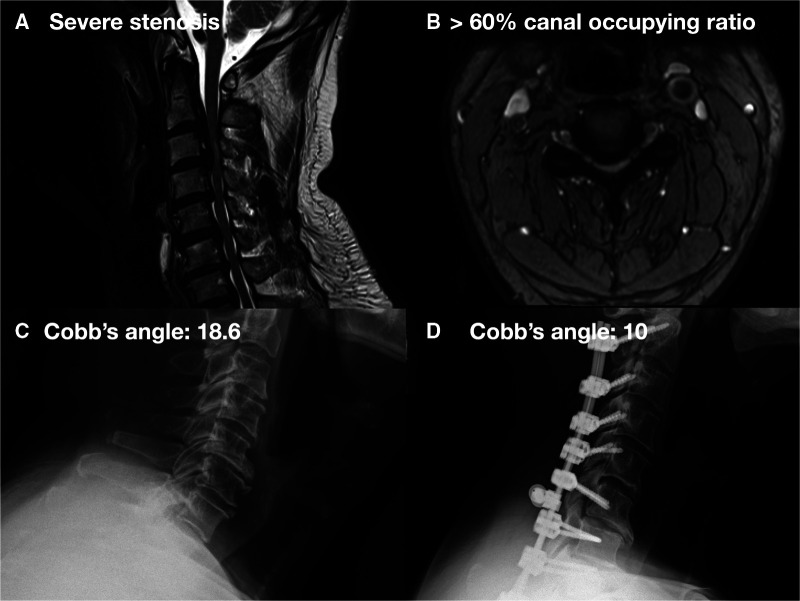
A 51-year-old male with CM due to long-segment massive OPLL over C2-7 underwent C2-7 laminectomy and C2–T2 fixation: (**A**) preoperative cervical MRI showed severe cervical stenosis with bulging disc. (**B**) Preoperative cervical MRI axial view showed >60% canal occupying ratio. (**C**) Preoperative cervical x-ray showed cervical lordosis about 18.6°. (**D**) Postoperative cervical x-ray showed cervical lordosis about 10°.

The study was approved by the Institutional Review Board (IRB: 202000602B0). Informed consent was waived due to the retrospective nature of this study. The OPLL diagnosis was confirmed by experienced radiologist. Inclusion criteria were (1) cervical compressive myelopathy with/without radiculopathy emanating from OPLL and involving more than three vertebrae, and (2) a maximal COR of more than 60% (11), which was defined as multilevel massive cervical OPLL (Figures 1, 2). Exclusion criteria included cases combined with fracture, significant congenital cervical anatomic deformity, or follow-up periods of less than 1 year. From January 2016 to December 2019, 55 patients (41 men and 14 women) were eligible for final analysis.

In our department, we managed patients with multilevel massive OPLL according to K-line (+) or (−) and whether involving the C2. On this view, the K-line was drawn from the midpoint of the spinal canal at C2 to the midpoint of the spinal canal at C7 (25). If K-line (+) and massive OPLL majorly involved the C2, posterior laminectomy above and below the OPLL-affected levels with posterior fixation and fusion with autograft bone harvested from the resected spinal process was done simultaneously (PDF), and facetectomy was done only done at fixation levels. If postoperative residual symptoms compared with inadequate decompression at canal or neuroforamen on a follow-up magnetic resonance imaging (MRI) were encountered, selective discectomy with interbody fusion and without fixation were performed. If K-line (−) occurred, laminectomy was performed at the compression level followed by corpectomy with or without discectomy, along with fixation and fusion (ADF) at one stage or on the day between postoperative first and second weeks. Corpectomy was done at major involving levels, but not more than two vertebral bodies, and then a wedge allograft was placed with autograft cancellous bone harvested from the resected vertebral bodies under distraction to create lordosis. For rigid or auto-fused segments with continuous OPLL or inadequate decompression by laminectomy in adjacent level(s), discectomy with removal of OPLL mass as much as possible with a high-speed burr for decompression was performed to break the OPLL mass. If anterior plate failure, screw loosening, graft subsidence, or dislodgement was noted at follow-up x-rays, posterior fixation and fusion with autograft bone harvested from the resected spinal process was done. Laminectomy followed by ADF or PDF depended on the decision of surgeons if K-line (+) or not majorly involving the C2.

Among these cases, 40 patients were treated with laminectomy followed by ADF, which was defined as group 1 (Figure 1), and 15 patients were treated with PDF, which was defined as group 2 (Figure 2). From these charts records, the following data were collected: name of the surgeon, patients’ demographics (age and gender), preoperative diagnosis, preoperative and postoperative images, decompression and fixation level, preoperative and postoperative visual analog scale (VAS) (26) and Japanese Orthopaedic Association Cervical Myelopathy Evaluation Questionnaire (JOACMEQ) (27, 28), and the amount of intraoperative blood loss. All surgeons were well trained and had at least 3 years of experience. In the preoperative and postoperative 1-year C2–C7 Cobb's angle on upright cervical spine lateral x-rays, the presence of cervical kyphosis (total curvature: straight: −4 to +4, lordosis: less than −4, kyphosis: more than +4) was measured (Figures 1, 2). Postoperative 1-year x-rays were also used to evaluate the position of the implant and whether graft subsidence occurred.

### Statistical analysis

The differences between the different groups were tested for significance using the Student’s *t* test for categorical variables. Univariate and multivariate analyses of clinical characteristics and outcomes were performed. Contingency statistics on categorical variables were performed with Fisher's exact test. All statistical tests were two-sided, and *p* < 0.05 was prospectively determined to establish statistical significance. All analyses were performed using IBM SPSS Statistics, version 25.

## Results

### Radiographic characteristics and postoperative outcomes

There were no significant demographic differences between the groups, as listed in Table 1. There was no statistical difference in the preoperative mean sagittal alignment degree (lordosis curve) (*p* = 0.549) and in the preoperative VAS score (*p* = 0.052). However, there was a significant difference in the postoperative cervical lordosis degree and postoperative 3-month VAS between the two groups (*p* = 0.003 and *p* < 0.001, respectively; Table 2). The JOACMEQ scores were not different in the two groups measured preoperatively or postoperatively (*p* = 0.369 and *p* = 0.062, respectively; Table 2). The degree of lordosis curve angle measured at postoperative 1-year C-spine lateral view in group 1 significantly improved compared with the degree measured preoperatively in group 1 (mean: 7.51 vs. 13.17, *p* < 0.001) and worse in group 2, but there was no significant difference (mean: 5.58 vs. 3.46, *p* < 0.176; Table 2). The postoperative VAS significantly improved than the preoperative VAS in the two groups (*p* < 0.001; Table 2). The postoperative JOACMEQ significantly improved than the preoperative one in group 1 (*p* < 0.001) and slightly worse than in group 2 (*p* = 0.164; Table 2).

**Table 1:** Demographic characteristics and radiographic characteristics of 55 patients with CM due to long-segment massive OPLL undergoing cervical operation.

	All *N* = 55	Group 1 *n* = 40	Group 2 *n* = 15	*P*-value
Sex				
Female	14 (25.5)	13 (32.5)	1(6.7)	0.292
Male	41 (74.5)	27 (67.5)	14 (93.3)	
Age, year	65.38	64.5	67.73	0.585
laminectomy level	4.35 (0.886)	4.38 (0.925)	4.27 (0.799)	0.69
Fixation level (SD)	4.15 (0.911)	4.10 (0.982)	4.27 (0.704)	0.551
VAS_ preop (SD)	6.51 (1.169)	6.33 (1.163)	7 (1.069)	0.052
JOACMEQ_ preop	13.93 (1.698)	13.8 (1.89)	14.27 (0.96)	0.237
K-line_ positive	47 (85.5)	32(80)	15 (100)	0.18
Blood loss, ml (SD)	238.36 (552.7)	177.25 (217.71)	401.33 (232.3)	0.405

Group 1: laminectomy and then ADF, Group 2: laminectomy and PDF.

CM, Cervical Myelopathy; OPLL, ossification of the posterior longitudinal ligament; VAS, Visual Analogue Scale; JOACMEQ, Japanese Orthopaedic Association Cervical Myelopathy Evaluation Questionnaire.

**Table 2:** Postoperative outcomes of 55 patients with CM due to long-segment massive OPLL undergoing cervical operation.

	Group 1 *n* = 40	Group 2 *n* = 15	*P*-value
Lordosis (degree) (SD)			
Preop	7.51 (9.9)	5.58 (10.76)	0.549
Postop_1y	13.17 (8.46)	3.46 (9.86)	**0.003**
*P*-value	**<0.001**	0.176	
VAS (1-10)			
Preop	6.33 (1.163)	7 (1.07)	0.052
Postop_3M	1.48 (0.751)	3 (1.13)	**<0.001**
*P*-value	**<0.001**	**<0.001**	
JOACMEQ			
Preop	13.8 (1.89)	14.27 (0.96)	0.369
Postop_3M	15.02 (1.42)	14.4 (0.91)	0.062
*P*-value	**<0.001**	0.164	

Group 1: laminectomy and then ADF, Group 2: laminectomy and PDF.

VAS, Visual Analogue Scale; JOACMEQ, Japanese Orthopaedic Association Cervical Myelopathy Evaluation Questionnaire.

### Factors related to cervical sagittal alignment

Tables 3, 4 show the results of univariate and multivariate analyses about the impact factors that contribute to cervical sagittal alignment including the clinical data, image signs, and way of approach. The improvement of postoperative cervical lordosis was not significantly associated with age, decompression level below C6 and involvement of C6, number of decompression level, and preoperative C2–C7 Cobb's angle in the univariate logistic regression analysis. On the other hand, the presence of a better cervical sagittal alignment was more likely associated with anterior fixation (OR: 28; 95% CI: 5.8–135.176; *p* < 0.001, univariate logistic regression). In the multivariable analysis, anterior fixation accounts for independent factors for better cervical sagittal alignment (Table 4).

**Table 3:** Predictors of better postoperative cervical lordosis in univariate analysis.

	CM due to long-segment massive OPLL (*n* = 55)			
Clinical data & Image signs	Lordosis worse* (*n* = 17)	Lordosis better* (*n* = 38)	OR (95% CI)	*P*-value*
Age, year	69.06 (9.424)	63.74 (9.983)	0.944 (0.886−1.006)	0.075
laminectomy below C6 level	15 (88.2)	34 (89.5)	1.133 (0.187−6.876)	0.892
laminectomy level number (SD)	4.35 (0.786)	4.34 (0.938)	0.986 (0.514−1.892)	0.966
Fixation level number (SD)	4.29 (0.686)	4.08 (0.997)	0.767 (0.405−1.454)	0.417
Group 1 (vs Group 2)	5 (29.4)	35 (92.1)	28 (5.8−135.176)	**<0.001**
K-line positive	15 (88.2)	32 (84.2)	0.711 (0.128−3.947)	0.697
Pre-op C2–C7 Cobb’s angle	8.812 (11.251)	6.174 (9.555)	0.973 (0.917−1.033)	0.369

Group 1: laminectomy and then ADF, Group 2: laminectomy and PDF.

* Lordosis was the comparison between preoperative and postoperative cervical X-ray.

**Table 4:** Multivariable analysis of better posterior cervical lordosis.

Independent factors	Odd ratio	95% CI	*P*-value
Age, year	0.937	0.856−1.026	0.16
laminectomy below C6 level	0.253	0.018−5.674	0.367
Fixation level number	0.831	0.322−2.144	0.701
Group 1 (vs Group 2)	24.695	3.619−168.492	**<0.001**

Group 1: laminectomy and then ADF, Group 2: laminectomy and PDF.

### Complications following two different approaches

The immediate postoperative and long-term complication rates are listed in Table 5; there are three patients (5.4%) with neurologic deficits and two patients (5%) in group 1, and one patient (6.7%) in group 2. One patient had posterior spinal cord epidural hematoma in group 1, who had salvage operation for removal of epidural hematoma. The patient had full recovery with no decrease in muscle power but still had some numbness as a preoperative condition over bilateral upper limb after 1 year rehabilitation. Two patients had postoperative four limbs muscle power decreased to grade 3 from grade 5 but no obvious somatosensory evoked potential change during intraoperative monitor, and they were highly suspected reperfusion of injury related after decompression (29), who gradually recovered to muscle power grade 4–5 after 3 months rehabilitation. Three patients (5.5%) had postoperative infection and two patients were in group 2. Two patients had gastrointestinal bleeding, which were highly suspected steroid related. In group 1, one patient had C5 palsy and another had mild graft subsidence at the 1-year follow-up and needed remedial surgery with posterior fixation and fusion. There were no patients with intraoperative shock, pulmonary embolism, cerebrospinal fluid (CSF) leakage, death, graft dislodgement, new onset subluxation, plate failure, or screws loosening during the 1-year follow-up.

**Table 5:** Postoperative complications of 55 patients with CM due to long-segment massive OPLL undergoing cervical operation.

	All *N* = 55	Group 1 *N* = 40	Group 2 *N* = 15
Neurologic deficit	3 (5.4)	2 (5)	1 (6.6)
EDH	1 (1.8)	1 (2.5)	0 (0)
Muscle edema	0 (0)	0 (0)	0 (0)
Reperfusion injury	2 (3.6)	1 (2.5)	1 (6.6)
Intraoperative Shock	0 (0)	0 (0)	0 (0)
Pulmonary embolism	0 (0)	0 (0)	0 (0)
Infection	3 (5.5)	1 (2.5)	2 (13.3)
Urinary tract infection	1 (1.8)	1 (2.5)	0 (0)
Pneumonia	1 (1.8)	0 (0)	1 (6.7)
Wound infection	1 (1.8)	0 (0)	1 (6.7)
GI bleeding	2 (3.6)	1 (2.5)	1 (6.7)
Death	0 (0)	0 (0)	0 (0)
Plate and screw loosen (1y follow up)	0 (0)	0 (0)	0 (0)
CSF leakage	0 (0)	0 (0)	0 (0)
C5 palsy	1 (1.8)	1 (2.5)	0 (0)
Graft subsidence (1y follow up)	1 (1.8)	1 (2.5)	0 (0)

Group 1: laminectomy and then ADF, Group 2: laminectomy and PDF.

EDH, epidural hematoma; GI bleeding, gastrointestinal bleeding; CSF, cerebrospinal fluid.

## Discussion

### Strategy for cervical myelopathy due to multilevel massive OPLL

The surgical treatment of multilevel massive OPLL is a highly controversial issue; previous studies have mainly focused on the comparison outcome and complication between ADF and PDF procedures (2–4, 7, 11–15, 18–20). Each approach has distinct advantages and disadvantages. Based on neurologic outcomes, ADF is considered for K-line (−) regardless of COR and K-line (+) and COR > 60% for patients with OPLL, which leads to a higher surgical trauma and incidence of surgery-related complications, such as CSF leakage, dysphagia, or hoarseness; PDF is considered for patients with multilevel compressive myelopathy, K-line (+), and COR < 50%–60% for patients with OPLL, which is relatively safer with lower surgical trauma and incidence of complications, such as C5 palsy and axial pain (3, 9, 14, 20, 21). The advantages of ADF are direct removal of offending pathologies from the front side of the dura and reconstruction of the anterior column of the cervical spine to maintain cervical lordosis and sagittal balance. In addition, ADF of more than two or three levels in patients with multiple massive OPLL would require additional posterior fusion with/without decompression to avoid graft-related complications such as dislodgement, subsidence, and pseudarthrosis (3, 24, 28).

A two-stage [posterior and anterior–posterior (*P*-AP) 540°] procedure could provide safe decompression, cervical realignment, and favorable outcomes for extensive cervical OPLL with kyphotic deformity (23). It is worth to concern about soft tissue and muscle damage *via* multiple *P*-AP approaches within 1-week period, such as dysphagia.

For those patients with multilevel massive OPLL or OPLL majorly involving the C2, our study proves that our protocol is safe, effective, and has few complications. So, we suggested the following:

1. If K-line (+) and multilevel massive OPLL majorly involves the C2, posterior laminectomy above and below the OPLL-affected levels with posterior fixation simultaneously is considered first.
2. For other multilevel massive OPLL, regardless of K-line (−) or (+), laminectomy at compression level followed by ADF depended on the severity and range of compression, but corpectomy of not more than two vertebral bodies on the day between postoperative first and second weeks is considered first.

Compared with PDF, our results showed that laminectomy and then ADF (corpectomy of two or less levels) for cervical multilevel massive OPLL resulted in a better postoperative cervical lordosis curve, less postoperative pain, and better JOACMEQ score, with equal postoperative complications.

### Postoperative outcome and complications

Compared with PDF (group 2), laminectomy followed by ADF (group 1) has the better postoperative 1-year lordosis curve, less axial pain, and better JOACMEQ score, with equal postoperative complications. All patients have better postoperative VAS than preoperative VAS in the two groups (*p* < 0.001), although postoperative cervical lordosis is worse compared to preoperative in group 2. However, the postoperative VAS in group 1 is significantly better than that of group 2 (*p* < 0.001), and it might be associated with the improvement in postoperative lordosis. Decompression of the neural elements could improve pain caused by myeloradiculopathy, and re-establishment of regional cervical alignment could improve axial neck pain. It had been reported that patients feel less neck tenderness after operation treated with ADF in CM with multilevel massive OPLL (4).

Neurologic deficits after laminectomy are the major complications including one patient with postoperative spinal epidural hematoma and two patients with reperfusion injury, which is characterized as unexplained new neurological deficits after an anterior or posterior decompressive cervical procedure and the radiographic hallmark is the presence of hyperintense T2 intramedullary signal change after a decompressive procedure without other pathologic changes (29). Plate or screws loosening, graft subsidence, or new onset subluxation at 1-year follow-up did not occur in any patient. Exceptions were minimal complications including three patients with infection and two patients with gastrointestinal bleeding, but they recovered well after medication. Both groups had a low complication rate.

### Factors related to cervical sagittal alignment

After anterior and posterior decompression, cervical sagittal alignment improved (23). Thus, we can easily manipulate and expand the disc space with retraction screws to gain more lordosis, and then insert the adequate expander or graft and fixation to treat cervical lordosis. On the other hand, PDF might limit the mobility and the extensible range due to head-pin fixation and limited decompression.

We also tried to seek other impact factors of cervical sagittal lordosis; however, only the procedure with posterior decompression followed by ADF (group 1) had a significant impact in our univariate logistic regression analysis and multivariable analysis. Factors such as “Preop C2–C7 Cobb's angle” have no influence on postoperative cervical lordosis. It could be explained by the fact that some kyphotic patients might gain more lordosis curve after the operation in our study. The similar concept on cervical myelopathy without OPLL patients was reported that preoperatively kyphotic patients benefitted more from surgery than lordotic patients (30).

## Limitation

Selection bias and lack of randomization could be anticipated in this retrospective study. Furthermore, the role of CM due to multiple massive OPLL is relatively strict and it limits our case number. A well-designed prospective validation in independent cohorts is needed to establish the ideal surgical strategy for multilevel massive and extensive OPLL.

## Conclusion

There is still no standard surgical guideline to manage cervical myelopathy due to long-segment massive OPLL, but we offer a safe and effective protocol. For patients with long-segment massive OPLL, regardless of the canal occupying ratio and K-line (−) or (+), we suggest laminectomy at compression level followed by ADF depending on the severity and range of compression or corpectomy of not more than three vertebral levels on the same day or within 2 weeks. For the patients with K-line (+) and massive OPLL involving the C2, we suggest posterior laminectomy above and below the OPLL-affected levels with posterior fixation simultaneously. In addition, we also demonstrated that cervical laminectomy followed by ADF could get enough decompression, minimalize the neurologic complication, get better cervical lordosis, and have less long-term axial pain in patients with CM due to long-segment massive OPLL.

## Data Availability

The raw data supporting the conclusions of this article will be made available by the authors, without undue reservation.
